# Qu-shi-hua-tan decoction's efficacy and safety for patients with angina following coronary revascularization: a randomized, double-blind, placebo-controlled trial study protocol

**DOI:** 10.3389/fcvm.2025.1512385

**Published:** 2025-04-30

**Authors:** Wenjing Xu, Jingwei Wen, Xijiu Li, Xiaoqing Li, Yan Zhang, Weihui Lu

**Affiliations:** ^1^ The Second Clinical Medical College of Guangzhou University of Chinese Medicine, Guangzhou, China; ^2^ State Key Laboratory of Traditional Chinese Medicine Syndrome, Guangdong Provincial Hospital of Chinese Medicine; ^3^ Guangzhou University of Chinese Medicine, Guangzhou, China; ^4^ Chinese Medicine Guangdong Laboratory, Guangzhou, Guangdong, China

**Keywords:** protocol, randomized controlled trial, Traditional Chinese Medicine, coronary heart disease, qu-shi-hua-tan decoction

## Abstract

**Introduction:**

The Qu-shi-hua-tan decoction (QSHTD), formulated by academician Chen Keji, is an empirical decoction for coronary heart disease (CHD). We conducted a randomized controlled trial to assess the effectiveness and safety of QSHTD in managing angina after coronary revascularization (AACR) in CHD patients.

**Methods and design:**

This double-blind randomized controlled trial will be conducted at Guangdong Provincial Hospital of Traditional Chinese Medicine. We will allocate 98 qualified participants to either the experimental or control group in a 1:1 ratio through random selection. The experimental group will be given standard care along with QSHTD, whereas the control group will receive standard care and a placebo. The study will span 26 weeks, consisting of a 2-week initial phase, a 12-week intervention phase, and a 12-week monitoring phase. The main outcome measure will be myocardial blood flow (MBF) assessed using adenosine stress real-time myocardial perfusion echocardiography (RTMPE). The secondary outcomes will be Canadian Cardiovascular Sociation Classification, Seattle Angina Questionnaire, Traditional Chinese Medicine (TCM) symptom evaluation; and major adverse cardiac events (MACE).

**Discussion:**

This study seeks to deliver compelling proof of the superior methodological and reporting standards of QSHTD's effectiveness and safety within AACR treatment.

**Clinical Trial Registration:**

Chinese Clinical Trial Registration Center [www.chictr.org.cn]. The trial was registered on November 26, 2020 [ChiCTR2000040270].

## 1 Introduction

Coronary heart disease (CHD) is a disease of vessel stenosis or occlusion due to coronary artery atherosclerosis. CHD can lead to myocardial ischemia, hypoxia or myocardial cell death. Among the global population, cardiovascular disease including CHD and stroke is the leading cause of death and disability (1). At present, coronary heart disease incidence among middle-aged and young people in China is 10.2%, and among the elderly it is 27.8%, with an increasing trend year-over-year (2). The widespread application of coronary artery revascularization is crucial to the treatment of CHD, and this includes percutaneous coronary intervention (PCI) and coronary artery bypass grafting (3). However, about 20%–40% of patients will experience persistent angina after coronary revascularization (AACR), which detracts from quality of life and incurs immense financial costs (4). Currently, there is no consensus on the mechanism of AACR. Ischemic etiologies such as stent restenosis and stent thrombosis, and functional causes including coronary spasm and microvascular dysfunction, have been noted as potential factors contributing to chest pain. Additional non-ischemic causes include psychological factors, stent tension, chest diseases, and gastrointestinal disease (5).

Existing research on the pathogenesis of postoperative angina following coronary artery bypass grafting is limited, with PCI being the focus. Oxidation, proinflammatory and anti-inflammatory imbalance, antioxidant dysfunction, severe vascular stenosis, low ejection fraction, microcirculation disorders, and diabetes have all been cited as risk factors for post persistent coronary intervention angina among the elderly (6–8). According to the guidelines, drug treatments include nitrates, beta blockers, calcium channel blockers, and ranolazine. Patients who require revascularization will undergo PCI or coronary artery bypass grafting treatment. However, for restenosis after PCI, although some patients can be resolved through PCI, many still refuse to undergo PCI. Moreover, modern medicine lacks efficient treatment for patients with coronary microvascular disease and diffuse lesions in grafts or PCI involving multiple vessels (9). Therefore, AACR remains a challenge (10).

Traditional Chinese Medicine (TCM) serves as a complementary and alternative treatment approach for AACR (11). According to clinical research results (12–14), the combination of TCM and standard treatment alleviates AACR, with a decrease in efficacy score. The levels of inflammatory markers including high sensitivity C-reactive protein, tumor necrosis factor-α, and interleukin-6 are also decreased., while blood lipids and hemorheology are improved. Additionally, TCM treatment can reduce the incidence of major adverse cardiac events (MACEs) in patients with CHD after PCI within 5 years (15). Research on TCM syndrome and medication analysis for CHD has shown phlegm-dampness syndrome to be one of the most important syndromes in the progression of the disease (16, 17).

Qu-shi-hua-tan decoction (QSHTD) is formulated based on the academician Chen Keji's clinical experience with CHD treatment, and has been widely used to treat AACR with phlegm-dampness syndrome (18). The decoction consists of Magnolia officinalis (Hou Pu), Rhizoma atractylodis (Cang Zhu), Pericarpium citri reticulatae (Chen Pi), Rhizoma pinellinae praeparata (Ban Xia), Curcuma zedoary (E Zhu) and Acorus tatarinowii (Shi Chang Pu) (Table 1). Our team's previous research by Taohua has indicated that QSHTD can improve cardiac function and reduce myocardial fibrosis in ApoE−/− mice after myocardial infarction and high-fat diet. It does this by regulating TLR4/NF-κb and Akt/HIF-1α signaling pathways (findings yet to be published). Shizhao utilized network pharmacology for an analysis which revealed that the small molecule compounds in QSHTD such as naringin and baicalin could stably bind to multiple gene receptors such as ESR1, AKT1 and MAPK1 (18).

**Table 1:** Components of qu-shi-hua-tan-decoction.

No.	Pinyin	Chinese	Latin scientific name	Scientific name	Part and form used	Place of origin (China)
1	Hou Po	厚朴	Magnolia officinalis	Magnolia officinalis Rehd. et Wils. var. biloba Rehd. et Wils.	Dried bark, root bark, or twig bark	Sichuan
2	Cang Zhu	苍术	Rhizoma atractylodis	Atractylodes chinensis (DC.) Koidz.	Dried rhizome	Jiangsu
3	Chen Pi	陈皮	Pericarpium citri reticulatae	Citrus reticulata Blanco	Dried tangerine peel	Guangdong
4	Ban Xia	半夏	Rhizoma Pinellinae Praeparata	Pinellia ternata (Thunb.) Breit.	Dried tuber	Sichuan
5	E Zhu	莪术	Curcuma zedoary	Curcuma kwangsiensis S. G. Lee et C. F. Ling	Dried rhizome	Guangxi
6	Schi Chang Pu	石菖蒲	Acorus tatarinowii	Acorus tatarinowii Schott	Dried rhizome	Sichuan

As of this writing, there is a notable deficiency in robust clinical data utilizing endpoint outcomes and adverse effects as evaluation metrics, which raises uncertainty regarding the efficacy and safety of QSHTD. To address this gap, we have designed a single-center, double-blind, randomized placebo-controlled trial to investigate the efficacy and safety of QSHTD in managing AACR associated with phlegm-dampness syndrome.

## 2 Methods

### 2.1 Design

**Figure 1:**
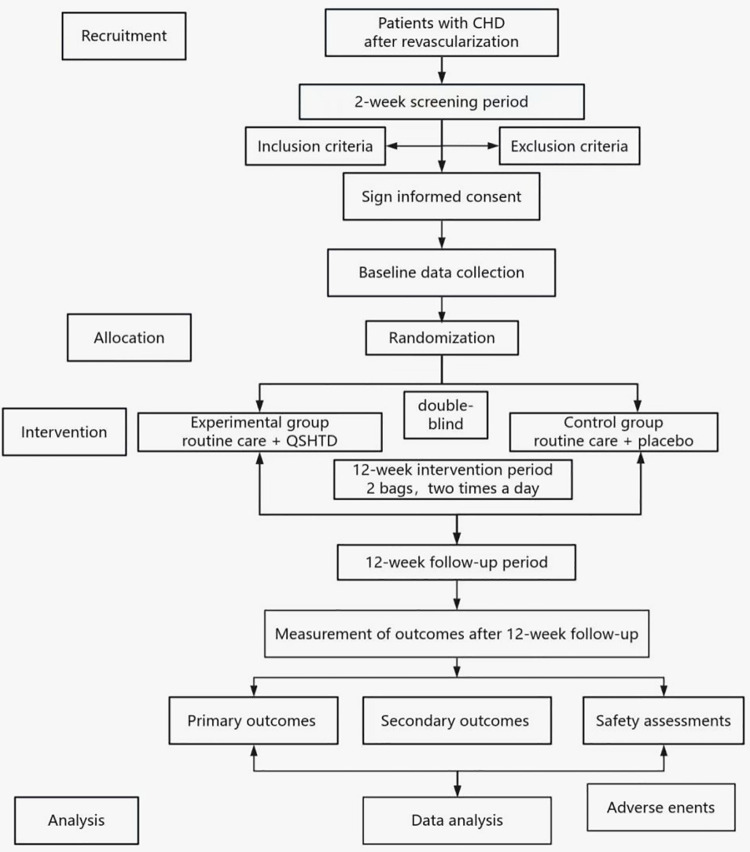
Study flowchart.

This is a prospective, randomized, double-blind, placebo-controlled trial. This trial was registered with the Chinese Clinical Trial Registry on November 26, 2020 (ChiCTR2000040270). This study was approved by the Research Ethics Committee at Guangdong Provincial Hospital of Traditional Chinese Medicine (BF2020-085-01). The trial complies with the Helsinki Declaration and the 2020 Good Clinical Practice guidelines. All 98 patients in this trial will be from Guangdong Provincial Hospital of Traditional Chinese Medicine. After they provide written informed consent, they will be randomly assigned to receive either QSHTD or a placebo. The study will consist of a 12-week intervention phase followed by a 12-week monitoring phase. A schematic representation of the study design is provided in Figure 1. The research process is listed in Table 2; it shows the recommended schedule for inclusion and follow-up as suggested by Standard Protocol Items: Recommendations for Interventional Trials (SPIRIT).

**Table 2:** Study procedure table.

Study phase time	Baseline	Intervention period			Follow-up
	−2∼0 weeks	4 weeks	8 weeks	12 weeks	24 weeks
Data collection at baseline					
Informed consent	√				
Inclusion/exclusion criteria	√				
Random number	√				
Demographic data	√				
Disease condition	√				
Medical history					
Vital signs	√			√	
Physical examination	√			√	
Medication situation	√	√	√	√	√
Laboratory testing and other tests					
Blood analysis	√			√	
Urine analysis	√			√	
Fecal analysis + OB	√			√	
Biological sample	√			√	
Electrocardiogram	√			√	
Echocardiography	√			√	
RTMPE	√			√	
Efﬁciency evaluation					
TCM symptom scores	√			√	√
CCSC	√			√	√
SAQ	√			√	√
Other data					
AEs	√	√	√	√	√
SAEs	√	√	√	√	√
Compliance	√	√	√	√	√
Complications	√	√	√	√	√
Clinical end-point events	√	√	√	√	√

OB, occult blood; HCY, homocysteine; HbA1c, glycated hemoglobin; hsCRP, high sensitivity C-reactive protein; MCE, myocardial contrast echocardiography; AEs, adverse events; SAEs, serious adverse events. “√” Represents the need for implementation at this point in time.

### 2.2 Participants

#### 2.2.1 Diagnostic criteria for CHD

According to the 2013 ESC guidelines on the management of stable coronary artery disease, if coronary angiography results show any coronary artery stenosis exceeding 50%, the patient has CHD (19).

#### 2.2.2 Diagnostic criteria for incomplete coronary revascularization

The calculation was based on the syntax score published in the *New England Journal of Medicine*, and residual syntax score (rSS) is the postoperative syntax score for the last revascularization surgery (20). rSS = 0 is defined as complete revascularization. rSS ≥ 1 is defined as incomplete coronary revascularization.

#### 2.2.3 Diagnostic criteria for TCM

The subjects must meet both the 2017 clinical diagnostic criteria for phlegm-damp syndrome in CHD (21) and the diagnostic criteria for dampness syndrome (22). Two certified TCM cardiologists will separately conduct syndrome differentiation based on the established diagnostic standards for TCM differentiation (Tables 3, 4).

**Table 3:** Diagnostic criteria for phlegm-damp syndrome.

Symptom type	Score	Item(s)
Main symptoms	3 points/item	1. Plump and teeth-printed tongue
		2. Greasy tongue fur
		3. Slippery tongue fur
Secondary symptoms	2 points/item	1. Oppression in chest
		2. Soft or slippery pulse
Other symptoms	1 points/item	1. Body trapped
		2. Sticky and greasy in mouth
		3. Obesity
		4. Viscous stool
		5. Distention and fullness
		6. Dim and dirty complexion
		7. Analeptic
		8. Anorexia

The diagnosis of phlegm dampness syndrome in coronary heart disease requires a cumulative score of ≥6 points on the indicators mentioned.

**Table 4:** Diagnostic criteria for dampness syndrome.

Indicator type		Content
Specific indicators	Category 1	1.1 Greasy tongue
		1.2 Slippery coating
		1.3 Thick coating
	Category 2	2.1 Head heaviness as if being wrapped up
		2.2 Body heaviness
		2.3 Limb heaviness
		2.4 Limited joint motion with heavy sensation
	Category 3	3.1 Sticky stool with non-smooth bowel movement
		3.2 Sticky and greasy sensation in mouth
		3.3 Excessive morbid leucorrhea/moist scrotum
		3.4 Greasy hair
Sensitivity indicators	Items	1. Obesity
		2. Somnolence
		3. Idleness and laziness
		4. Non-smooth hidrosis
		5. Dirty face
		6. Dizziness
		7. Fullness and oppression in chest
		8. Excessive phlegm
		9. Distention and fullness in the epigastric region
		10. Lower abdominal fullness
		11. Soreness and weakness in the waist and knees
		12. Soreness in the joints and muscles
		13. Absence of thirst or thirst without desire to drink
		14. Poor appetite
		15. Loose stool
		16. Fetid mouth odor
		17. Enlarged tongue
		18. Soft pulse
		19. Slippery pulse

The diagnostic principle of dampness syndrome: If any one of the following conditions is met, dampness syndrome can be diagnosed: ① Meeting any one category of a specific indicator. ② Having three sensitivity indicator items. Note 1: Specific indicators are measured in category units; sensitivity indicators are measured in item units. Note 2: Specific indicators belonging to the same category, whether there is only one or multiple at the same time, are counted as one specific indicator of the same category.

#### 2.2.4 Inclusion criteria

(1) Age range is from 18 to 80 years old.
(2) PCI performed within the previous 2 years and rSS ≥ 2 points; or PCI was performed at least 2 years before and coronary angiography was performed within the previous 2 years with rSS ≥ 2 points; or PCI was performed at least 2 years before and there is untreated or unsuccessfully treated chronic total occlusion.
(3) Meet the 2017 clinical diagnostic criteria for phlegm-damp syndrome in CHD, as well as the dampness syndrome diagnostic criteria.
(4) Agree to undergo adenosine stress real-time myocardial perfusion echocardiography (RTMPE).
(5) A signed consent form is required.

#### 2.2.5 Exclusion criteria

(1) Cardiogenic shock.
(2) Severe heart failure (New York Heart Association classiﬁcation stages II–IV, or left ventricular ejection fraction ≤ 50%), or comorbid with severe valvular disease.
(3) Severe liver or kidney dysfunction (serum alanine aminotransferase of at least double the normal upper limit and/or serum creatinine ≥ 265 μmol/L).
(4) Patients with other vascular diseases, such as cerebrovascular disease, arteriosclerosis obliterans of the lower limbs or diabetes nephropathy.
(5) Patients with active bleeding or severe hematopoietic system disease.
(6) Patients with malignant tumors who are undergoing chemotherapy or radiotherapy who have not been clinically cured, or those who have been treated with special drugs for malignant tumors, or with life expectancy under 3 years.
(7) Taken antibiotics within the previous 2 weeks.
(8) Pregnant (or preparing to become pregnant) women, lactating women.
(9) Have participated in, or are participating in, other clinical trials within the past 3 months.
(10) The researcher determined that the participant was unable to finish the study or adhere to its requirements.

### 2.3 Recruitment process

The research will include participants from both the outpatient and inpatient departments of the Guangdong Provincial Hospital of Traditional Chinese Medicine. Subjects who meet the eligibility criteria and provide informed consent will advance to a screening phase, while those who do not fulfill these requirements will be excluded prior to randomization. Principal investigators and attending physicians will jointly conduct eligibility assessment for enrolled participants based on specific inclusion/exclusion criteria. Registered participants will then be allocated through an online system for intervention and follow-up phases of the experiment. Ineligible individuals' demographic data, as well as any reasons for non-participation, will also be documented alongside strict confidentiality measures.

### 2.4 Randomization and allocation concealment

The randomization process will employ a block randomization method. 98 patients will be randomly allocated into two groups at a ratio of 1:1 using block randomization. Each block will consist of 49 cases with random sequence arrangements within the blocks. We plan to use the PROC PLAN procedure in SAS V9.4 for this study. The results of both the randomization and the allocation procedures were conducted by researchers from the Clinical Research Methodology Team at Guangdong Provincial Hospital of Traditional Chinese Medicine. The random assignment procedure was registered with the TCM Clinical Research Methodology Team at Guangdong Provincial Hospital of Traditional Chinese Medicine. Random assignment results will be generated and released via a centralized randomization management system. Researchers will log into an online randomization system to apply for randomization and receive the randomization results.

### 2.5 Blinding

This trial will be conducted in a double-blind manner, ensuring that neither the participants nor the research staff are aware of the group assignments. The Jiangyin Tianjiang Pharmaceutical Co., Ltd. (Jiangyin, China) will produce both the QSHTD and the placebo granules. The placebo will be designed to closely resemble the QSHTD in all aspects, such as color and flavor. All medications will adhere to the standards set by the National Drug Manufacturing Code of China. The blinding process and the maintenance of the blind will be handled by the TCM Clinical Research Methodology Team at Guangdong Provincial Hospital of Traditional Chinese Medicine. An emergency letter for each drug code will be created during the randomization and blinding process, and it will be securely sealed according to the corresponding drug group. If a serious adverse event (SAE) occurs or if urgent medical care is required, the principal investigator can use the online randomization system to request unblinding. When unblinding becomes inevitable, the participant will be excluded from the study, and the investigator must inform the examiner of the cause within 24 h. If unblinding is essential, the individual will be removed from the research, and the investigator must convey the reasons to the examiner within a 24-hour period.

### 2.6 Intervention

Referring to the 2018 ESC Guidelines for Coronary Artery Revascularization, all subjects will be given antiplatelet drugs, statin lipid-lowering drugs, nitrate esters, anticoagulants, and *β* receptor blocker (23). After a subject is enrolled, their previous standard treatment plan will be maintained. Any modifications to the treatment, the patient's status, or the drug's quantity or variety should be documented in details.

#### 2.6.1 Experimental group

The participants in the experimental group will receive standard care along with QSHTD (two 7.4 g packets, taken orally twice daily after meals) for a duration of 12 weeks, and the entire observation and follow-up period will span 24 weeks.

#### 2.6.2 Control group

The control group participants will receive standard care along with placebo granules (two 7.4 g packets, taken orally twice daily after meals) for a duration of 12 weeks. The entire observation and follow-up period will span 24 weeks.

#### 2.6.3 Concomitant medication during the trial

(1) Use of any other traditional Chinese patent medicines or decoctions will be prohibited during the test.
(2) Any patients entering the screening period must discontinue the usage of any long-acting nitrate drugs (such as long-acting nitroglycerin) or any other anti-angina treatments. However, they may continue using a beta-blocker and/or calcium channel blocker if they are taking one.
(3) Throughout the study period, patients will be prohibited from adding or using any of the following medications, in addition to maintaining their original medication: beta blockers, calcium antagonists, nitrate drugs (except for the use of short-acting nitroglycerin for angina attacks).
(4) Any medications or treatments necessary for managing coexisting conditions should be documented, including the drug name (or other treatment name), dosage, frequency of use, and duration. This data is crucial for both analysis and reporting.

### 2.7 Outcome measurements

#### 2.7.1 Primary outcomes

The main endpoint is the change in myocardial blood flow (MBF), assessed through adenosine stress RTMPE, following a 12-week treatment period.

#### 2.7.2 Secondary outcomes

(1) Canadian Cardiovascular Society Classification (24); (2) Seattle Angina Questionnaire (24); (3) TCM symptom evaluation; (4) MACE incidence rate, including all-cause mortality, nonfatal myocardial infarction, stroke, target vessel revascularization and stent thrombosis events.

Table 2 delineates the precise collection times for the outcome measures.

### 2.8 Safety assessment

Our team will meticulously document the exact timing, intensity, duration, interventions applied, and potential outcomes of all adverse incidents. For each documented incident, the investigator will also assess the connection between the adverse events and the medications under study. In line with ICH guidelines, SAEs will be classified as any occurrences that could lead to hospitalization, inability to work, birth defects, or fatalities (25). The investigator will notify the ethics committee of any SAE in 24 h.

### 2.9 Sample size calculation

The sample size calculation will be based on the mean and standard deviation of the variation in MBF. According to the literature, the MBF variation in a conventional treatment group increased by 0.52 ± 0.72 (dB/s) after 12 weeks, while in the conventional combined TCM treatment group, MBF variation increased by 0.97 ± 0.68 (dB/s) after 12 weeks (26). After substituting the above values into PASS11 software with *α* = 0.05 and *β* = 0.20, the sample size required for each group was determined to be 39 cases. Accounting for a 20% dropout rate, this was adjusted to 49 cases per group. The two groups required a total of 98 patients.

### 2.10 Data analysis and managament

Data analysis will be performed using SAS 9.2 (SAS Institute Inc., USA) according to a pre-established statistical plan. The evaluation of effectiveness will adhere to the intention-to-treat principle. Quantitative data will be analyzed using methods such as ANOVA. Categorical data will be evaluated with chi-square tests and Fisher's exact test, while non-parametric rank sum tests and CMH chi-square tests will be used for ordinal data. The significance level will be set at *α* = 0.05, and a *P*-value below 0.05 will indicate a statistically significant difference. Descriptive statistics will summarize the baseline characteristics of the two groups. For continuous variables that are normally distributed or have an unknown distribution, *T*-tests or Mann–Whitney *U*-tests will be employed. To address missing data, appropriate imputation strategies will be used to minimize potential bias. This will involve multiple imputation techniques, allowing us to create several complete datasets by estimating missing values based on observed data trends. Proper handling of data is crucial for maintaining the precision, reliability, and traceability of clinical trial information, as stipulated by the Technical Guidelines for Clinical Trial Data Management (27) and the Good Clinical Practice standards (28).

### 2.11 Quality control

To guarantee the enforcement of quality control and assurance in clinical research, the researchers will carry out their responsibilities, adhere to the clinical trial protocol, utilize standard operating procedures, and validate all pertinent observations and results. In these studies, the allocation of participants must be based on the study design. For a randomized assignment, the statistical unit will determine each participant's treatment group, and this information will be kept confidential from the researchers. Researchers are required to provide essential training to all personnel involved in the clinical trial, disseminate relevant data, operational procedures, and/or roles, and ensure that the data is reliable, precise, comprehensive, and timely. This data must also be legally documented in medical records and CRFs, which should be stored securely. Monitors will conduct systematic reviews of the activities and materials pertaining to clinical trials must be evaluated for adherence to the study protocol, standard operating procedures, and pertinent legal and regulatory requirements. It is essential that all clinical laboratory results are documented, with a duplicate of the initial report appended to the CRFs. The clinical investigation will follow the sanctioned protocol, with any deviations from it being documented. Any adjustments to the study protocol must be detailed and submitted to the ethics committee for authorization before being enacted. Participants adhering to the regimen for 80% or more of the prescribed time will be considered highly compliant. All non-adherence instances and their causes will be documented.

### 2.12 Participant safety

Physicians participating in clinical trials must provide subjects with detailed information about the clinical trial, so that they can comprehend and voluntarily sign an informed consent form before the clinical trial commences. Participants are free to exit the trial at any point and for any cause; signing the informed consent document and meeting the eligibility criteria will not impact their future medical care. Although individuals have the freedom to exit the study whenever they wish and for any reason, it is advisable to refrain from doing so unless there is a significant necessity. Additionally, measures should be undertaken to complete as much follow-up as possible. This will enable the evaluation of its efficacy and safety.

## 3 Discussion

QSHTD, a formula developed by academician Chen Keji, has been widely used in managing angina. This study stands out as the first high-quality, randomized, double-blind, placebo-controlled trial in China to offer robust clinical evidence for enhancing treatment strategies for CHD.

Multiple methods have been studied for recurrent and persistent AACR; however, the overall effect has not been promising (5). Research has shown that the efficacy of the combination of TCM and standard treatment is superior to that of only standard treatment (29). However, existing research on the TCM treatment of CHD has focused on blood stasis syndrome, and has been based on either promoting blood circulation and removing stasis or supplementing qi and promoting blood circulation. There have been few treatments for phlegm and dampness.

Previous studies have reported that phlegm dampness syndrome has a high prevalence in CHD patients (30, 31). “Dampness”, “phlegm”, and “stasis” are the key pathological products of CHD. They are also factors underlying the prolongation, progression, and deterioration of the disease. When treating CHD with TCM, Chen Keji emphasized eliminating dampness and dissipating phlegm as being as critical as promoting blood circulation and removing blood stasis. Furthermore, multiple studies have shown that the combination of dampness elimination and phlegm dissipation with standard treatment can diminish the restenosis rate of coronary artery soft plaque after PCI, lower blood lipid levels, lower high sensitivity C-reactive protein, inhibit platelet aggregation, and reduce angina symptoms. Moreover, Gualou Xiebai granules combined with conventional Western medicine can diminish the PCI postoperative restenosis incidence rate in soft plaque in the coronary arteries with phlegm dampness blocking syndrome, reduce blood lipid levels and improve clinical symptoms (32). Additionally, Dan Lou tablets improve the TCM main syndrome scores of phlegm and blood stasis syndrome in patients with CHD after PCI (33). Moreover, Huazhuo Qushi Tongxin decoction can improve clinical symptoms and serum inflammatory factor levels, and improve patients' cardiac function (34). Tongmai Jiangzhuo granules also can relieve patients' angina symptoms after coronary revascularization, improve the TCM syndrome of phlegm, turbidity and blood stasis, and improve the life quality prognosis (35).

MBF represents the blood volume flowing through a specific cardiac mass per unit of time (ml/min/g) (36). In this study, we will use RTMPE to assess MBF. Image analysis for quantiﬁcation will be conducted with software (QLAB version 6.0; Phillips Medical System). The software will automatically determine the signal intensities (in decibels) for both the myocardial plateau and the neighboring left ventricular plateau, along with the signal intensity transfer rate (*β*; per second). The normalized myocardial acoustic intensity (An) is calculated as the ratio of the myocardial plateau signal intensity to the signal intensity of the adjacent left ventricular plateau. The product of An and *β* represents MBF (dB/s) (37). Canadian Cardiovascular Society Classification will be used to define physician-reported angina (24). The Seattle Angina Questionnaire, a patient reported functional status measure that is designed speciﬁcally for CHD, will be used to assess CHD patients' functional status (24).

Unlike previous which have entailed the use of real-time myocardial contrast-enhanced echocardiography, in our trial we used adenosine stress RTMPE. The quantiﬁcation of MBF during adenosine stress RTMPE is the primary outcome. The current noninvasive gold standard for diagnosing myocardial ischemia involves assessing MBF and coronary flow reserve using positron emission tomography. However, radionuclide imaging techniques are expensive and often unfeasible. Furthermore, there is a high correlation between myocardial acoustic contrast technology and positron emission tomography measurement of MBF (38). The main advantages of adenosine stress RTMPE are that there is no ionizing radiation, it is easy to operate, and it is cost-effective (39). In adenosine stress RTMPE, the application of quantitative analysis methods to evaluate MBF reserve in CHD patients is more accurate and has important value in guiding clinical prognosis. Abnormal MBF reserve and abnormal *β* reserve were indicators of overall and primary outcomes, such as cardiac mortality, non-fatal heart attacks, and intractable or unstable angina necessitating coronary revascularization during the same hospital stay, provided that elective coronary revascularization occurs at least 3 months after RTMPE (37).

Both the Canadian Cardiovascular Sociation Classification and the Seattle Angina Questionnaire have been widely used to evaluate angina severity and quality of life (24). MACE incidence rate is used to evaluate drugs' effects on the prognosis of patients with coronary heart disease. In addition, in the systematic evaluation of TCM treatment for CHD and angina pectoris, the assessment of TCM symptoms frequently acts as the benchmark for measuring clinical effectiveness (40). Moreover, tracking adverse events and reactions is crucial for assessing drug performance and for directing clinical medication practices (41).

This study's potential shortcomings should not be ignored. Even though our total follow-up period was 24 weeks, which is longer than other similar clinical studies, we may have been unable to observe major adverse cardiovascular disease events. In addition, this is a single center study, and there may be regional variation in the results. Additionally, the quality of the adenosine stress RTMPE images depends on the level of operation, and is also influenced by factors such as obesity, respiratory movement, and lung disease.

In conclusion, this study will deliver robust data supporting QSHTD as an integrated treatment for AACR. Regardless of whether the results are positive or negative, they will possess significant clinical implications for patients.
